# Role of Suppressor Variables in Primary Prevention Obesity Research: Examples from Two Predictive Models

**DOI:** 10.1155/2014/567523

**Published:** 2014-01-30

**Authors:** Adam P. Knowlden

**Affiliations:** Department of Health Science, University of Alabama, Russell Hall 472, P.O. Box 870311, Tuscaloosa, AL 35487-0311, USA

## Abstract

Pediatric obesity is a pertinent public health challenge. Child physical activity and screen time behaviors enacted within the context of the family and home environment are important determinants of pediatric obesity. The purpose of this study was to operationalize five, maternal-facilitated, social cognitive theory constructs for predicting physical activity and screen time behaviors in children. A secondary purpose was to elucidate the function of suppressor variables in the design and implementation of family- and home-based interventions seeking to prevent pediatric obesity. Instrumentation included face and content validity of the measurement tool by a panel of experts, test-retest reliability of the theoretical constructs, and predictive validity of the constructs through structural equation modeling. Physical activity and screen time were modeled separately according to the five selected social cognitive theory constructs. Data were collected from 224 mothers with children between four and six years of age. Specification indices indicated satisfactory fit for the final physical activity and screen time models. Through a series of four procedures, the structural models identified emotional coping and expectations as suppressor variables for self-efficacy. Suppressor variables can complement program design recommendations by providing a suggested ordering to construct integration within an intervention.

## 1. Introduction

Pediatric obesity impacts children worldwide and remains a formidable public health challenge [1]. Elucidating theoretical determinants of pediatric obesity is necessary for the development of primary prevention interventions that can reduce obesity prevalence [2]. Two activity-based behaviors posited to contribute to pediatric obesity include physical activity and screen time [3]. Physical activity is recognized as a modifiable determinant in the prevention of noncommunicable diseases including cardiovascular disease, hypertension, type 2 diabetes mellitus, and obesity [4]. Reduced levels of physical activity increase the likelihood of pediatric obesity. The Framingham Children's Study found that preschool children with lower levels of physical activity gained significantly more subcutaneous fat than did active children [5]. A three-year longitudinal study of preschool children found that increases in leisure physical activity and higher levels of aerobic activity led to decreased body mass index [6]. Recommendations for children include a total of 60 minutes of physical activity each day [3, 7]. Screen time is a primary source of sedentary activity for children and a recognized risk factor for pediatric obesity [8]. Screen time includes leisure time spent in front of a television or computer screen [7]. It is generally recommended that children receive no more than 120 minutes of screen time each day [3, 7].

From a psychosocial perspective, researchers are increasingly recognizing the influence of the family and home environment on factors such as physical activity and screen time that contribute to pediatric obesity [9]. However, there is a dearth of behavioral prediction model research for designing and measuring family- and home-based interventions targeting these behaviors [10]. Social cognitive theory (SCT) is a robust, theoretical framework for eliciting health behavior change [11]. SCT is based on the premise of reciprocal determinism; a casual paradigm that posits that human functioning is the result of environmental, personal, and behavioral factors. SCT is rooted in human potential and emphasizes modeling, symbolizing, forethought, self-regulatory, and self-efficacy capabilities. SCT was selected as the theoretical framework for this study for three primary purposes. First, it was the theory applied most frequently (*n* = 2) in a systematic review examining family- and home-based pediatric obesity interventions [10]. Second, although SCT is considered an interpersonal-level behavior change theory, it allows for the reification of the environment, which is beneficial for targeting the home milieu. Finally, SCT is grounded in vicarious learning. Evidence suggests that children develop physical activity attitudes and behaviors primarily through observation of parental role models [9].

The five SCT variables operationalized in this investigation included environment, emotional coping, expectations, self-control, and self-efficacy. The constructs operationalized in this study have been applied throughout various streams of pediatric obesity research and have demonstrated their usefulness in interventions targeting behavior change [12]. In terms of SCT, the environment contains both the social and physical milieu [13]. Environment was selected, as availability and accessibility to physical activity and screen time are important mediators of engagement in these behaviors [14]. These factors are even more pronounced in family- and home-based pediatric interventions, as parents are the foremost role models from whom young children develop activity preferences [9]. Self-efficacy has been identified by Bandura [15] as the most salient determinant of behavior change within SCT. Self-control works in tandem with self-efficacy to promote behavior change and was considered important for actualizing self-efficacy [2]. Sharma et al. [16] identified self-efficacy as a primary predictor for daily physical activity among fifth grade school children. The study [16] also found that self-control was associated with reduction of screen time exposure. Expectations perform an integral role in motivation to modify a behavior and were considered a core construct of SCT [17]. Prior research has identified a dose-response relationship between expectations and physical activity [18]. Emotional coping was deemed important in predicting the targeted behaviors, as young children often initially reject behavior modification [19]. To compensate, parents may use authoritarian parenting techniques, which can produce negative long-term outcomes on a child's perceptions of health behaviors [20]; therefore, it was considered advantageous to provide participants with skills and techniques to reduce stress associated with health behavior change in children. Based on prior family- and home-based pediatric obesity research, mothers were selected as the target population for this investigation [8, 21–24]. Children ages 4 to 6 year were targeted for this study as this age range has been demonstrated to be a strong predictor of future health [8, 25].

Given this backdrop, the purpose of this study was to begin addressing the scarcity of behavioral prediction models required to advance family- and home-based, pediatric obesity, primary prevention intervention research. To accomplish this objective, five SCT maternal-facilitated constructs (environment, expectations, emotional coping, self-control, and self-efficacy) hypothesized to predict physical activity and screen time behaviors in children were operationalized and modeled. The behaviors were modeled separately according to the five SCT constructs. Subsequently, this study reports findings of two models. Model one tests the reliability and validity of the five SCT constructs regressed on physical activity. Model two tests the reliability and validity of the five SCT constructs regressed on screen time. As elucidated in the results section of this report, some theoretical constructs entered into the models were identified as suppressor variables. Suppressor variables control for error variance in one or more independent variables within a regression model. Therefore, a secondary objective of this report was to demonstrate the role of suppressor variables in primary prevention, pediatric obesity research, specifically, to explain how suppressor variables can help to inform intervention design and implementation.

## 2. Materials and Methods

### 2.1. Participants

Sample size for model building was established using Westland's [26] structural equation modeling calculator. Respondents included convenience samples of mothers recruited from a large, north American Midwestern University, a large, north American Midwestern Hospital, childcare centers located throughout the Midwest region of north America, as well as a preestablished subject pool.

### 2.2. Instrumentation

Instrumentation encompassed three stages of data collection and analysis. Stage one evaluated face and content validity of the instrument through a panel of six experts over a two-round review process. Stage two evaluated test-retest reliability of the instrument. Stage three evaluated construct and predictive validity of the instrument through structural equation modeling.

### 2.3. Stage One Procedures

The initial instrument items were adapted from a previous validated instrument developed by Sharma et al. [16] that examined SCT, physical activity, and screen time predictors of pediatric obesity within a school-based context. The original, self-report instrument was designed to be completed by school children; thus the items were modified for relevancy to mothers with young children. After the initial items were created, a panel of experts was formed. The panel of experts consisted of two pediatric obesity experts, two health behavior experts and two SCT experts. Each panel member was sent a cover letter explaining the purpose of the study, a copy of the draft instrument, and a feedback assessment form to evaluate face and content validity of the instrument. Members were asked to provide a general critique of the instrument and to make suggestions for improvement based upon their individual areas of expertise. Feedback materials were requested to be returned within a two-week time frame. Modifications were made to the instrument based on the jurors' feedback. After incorporating the recommended amendments suggested by the panel, a modified version of the instrument was delivered to the panel for a second round of review. For round two, jurors received a summarized list of all changes made to the instrument, a copy of the revised instrument, and an instrument assessment form. Jurors were requested to submit round two feedback within a two-week period. Once again, panel member recommendations were incorporated. Unanimous agreement concerning the face and content validity of the instrument was reached after round two; thus the panel of experts was disbanded. The final, 40-item instrument was organized into four parts: (1) inclusion/exclusion criteria for participation, (2) 24 hour, maternal-proxy behavioral recall of child physical activity and screen time behaviors, (3) operationalized, maternal-facilitated constructs of SCT, and (4) demographic information.

Part one of the instrument applied inclusion criteria to gauge participant eligibility. For the purposes of this study, eligibility was limited to English-speaking mothers with at least one child in the age range of 4 to 6 years. Exclusion criteria included pregnant mothers, mothers with a child inside the age range of 4 to 6 years with a disability that would interfere with participating in physical activity, a child with a medical condition associated with weight gain, a child prescribed medication associated with weight gain, or a child currently enrolled in an additional weight-management program. Participants were requested to either agree or disagree with each criterion. Those participants unable to satisfy inclusion/exclusion criteria were ineligible to participate. For eligible participants with more than one child in the age range of 4 to 6 years, the respondent was requested to answer the instrument items with their oldest child in mind.

Part two of the instrument assessed the physical activity and screen time behaviors of the participants' children. The physical activity item requested “Yesterday, how many total minutes was your child physically active throughout the day?” Screen time items queried “Yesterday, how many minutes did your child watch TV, DVDs, or movies”, “Yesterday how many minutes did your child spend on a computer?”, and “How many minutes did your child spend playing video games?” Participants were requested to respond in short answer format by indicating the total quantity of the given behavioral unit in the previous 24 hours. Illustrated examples of each behavior were provided on the instrument to assist comprehension of the requested information.

Part three of the instrument comprised the scales for measuring the maternal-facilitated SCT constructs for child physical activity and screen time behaviors. Both models included three items to assess the home environment of the mother's child to engage in the physical activity and screen time behaviors. For both models, the score range for the environment construct was 3 to 15. Higher scores implied an environment more conducive to increasing physical activity and reducing screen time. For both models, the stem statement “How often do you” was followed by 5-point, Likert-type scales with endpoints ranging from “never” to “always.” Sample scale items from model 1 included “participate in physical activity in front of your child in your home, yard, or apartment complex?” and “find fun ways for your child to be physically active in your home, yard, or apartment complex?” Sample scale items from model 2 included “find alternatives to screen time at home for your child?” and “participate in screen time at home with your child?”

Both models included three items to measure the capacity of the mother to assist her child with adjusting emotionally and psychologically to increasing physical activity and reducing screen time. For both models, the score range for the environment construct was 3 to 15. Higher scores indicated stronger emotional coping capabilities of the mother to encourage physical activity and to reduce screen time. For both models, the emotional coping stem statement “How sure are you that you can help your child” was proceeded by 5-point, Likert-type scales with endpoints ranging from “not at all sure” to “completely sure.” Sample scale items from model 1 included “adjust to being physically active for 60 minutes each day?” and “manage any negative emotions from them if you encourage them to be physically active?” Sample scale items from model 2 included “manage any negative emotions from them while reducing screen time?” and “adjust to reducing screen time to less than 2 hours per day?”

Expectations were comprised of the multiplicative score of two subconstructs: outcome expectations and outcome expectancies. Both models included four items to measure outcome expectations and four complementary items to measure outcome expectancies of the two behaviors. The summative scores of the outcome expectations and outcome expectancies constructs were multiplied for a potential product score range of 4 to 100. Higher scores indicated stronger maternal expectations regarding engagement in physical activity and refrainment of screen time. For both models, the outcome expectations stem statement “If your child participates in physical activity/screen time every day they will” was proceeded by 5-point, Likert-type scales with endpoints ranging from “not at all likely” to “extremely likely.” Sample scale items for both models included “have a better weight?”, and “have more energy?” For both models, the outcome expectancies stem statement, “Overall, how important is it to you that your child will” was proceeded by a 5-point, Likert-type scale with endpoints ranging from “not at all important” to “extremely important.” Sample scale items for both models included “have more self-confidence?” and “be more relaxed?”

Both models included three items to measure the capacity of the mother to set goals to increase physical activity and decrease screen time. For both models, the score range for the self-control construct was 3 to 15. Higher scores indicated stronger self-regulatory abilities of the mother to promote physical activity and discourage screen time. For both models, the self-control stem statement “How sure are you that you can” was proceeded by 5-point, Likert-type scales with endpoints ranging from “not at all sure” to “completely sure.” Sample scale items from model 1 included “reward your child with something they like for being physically active for 60 minutes every day?” and “plan to have your child be physically active for 60 minutes every day?” Sample scale items from model 2 included “set goals to reduce your child's screen time to no more than 2 hours per day?” and “reward your child for reducing screen time?”

Both models included three items to measure the confidence of the mother to increase physical activity and reduce screen time. For both models, the possible score range for the self-efficacy construct was 3 to 15. Higher scores indicated robust self-efficacy capabilities of the mother to increase physical activity and reduce screen time. For both models, self-efficacy stem statement “How confident are you that you can get your child to” was proceeded by 5-point, Likert-type scales with endpoints ranging from “not at all confident” to “completely confident.” Sample scale items from model 1 included “physically active for 60 minutes every day?” and “physically active for 60 minutes every day, even if you are tired?” Sample scale items from model 2 included, “spend no more than 2 hours per day with screen time?” and “reduce screen time even if their favorite show is coming on?”

The final section of the instrument was devoted to collecting demographic data. Multiple choice questions were used to obtain race/ethnicity of the mother, marital status of the mother, sex of the child, and race/ethnicity of the child. Self-reported ages of the participant and the child were acquired using short answer format.

### 2.4. Stage Two and Three Procedures

For stage two, test-retest reliability coefficients were calculated for both exogenous and endogenous variables to assess stability over time. In conducting test-retest analysis, 30 volunteering participants were requested to complete the instrument two times, with two weeks between administrations of the instrument. Acceptable test-retest Pearson's *r* test-retest coefficient values were set *a priori* at 0.70 [27]. For stage three, Kline's [28] two-step modeling procedure was applied to specify each behavioral model. Confirmatory factor analysis (CFA) was conducted to assess model fit. CFA was evaluated through the model chi-square test (*χ* ^2^) with significance testing set *a priori* at *P* value greater than 0.05 and root mean square error of approximation (RMSEA) less than 1.00 [29]. Additional fit indexes included the goodness-of-fit index (GFI), adjusted goodness-of-fit index (AGFI), normed fit index (NFI), and comparative fit index (CFI), all of which should exceed 0.90 by convention [28]. Convergent validity assessed the extent to which the model indicators of each construct converged adequately. In assessing convergent validity, indicator factor loadings, construct reliability, and average variance extracted were calculated [30]. Factor loading values less than 0.50 were considered for removal. In maintaining conventional standards, average variance extracted was set *a priori* at no less than 0.50 and construct reliability was set at no less than 0.60 [30].

Following development of the measurement models, structural models were built to determine the variance explained in physical activity and screen time behaviors by the maternal-facilitated SCT variables. Significance levels for direct path coefficients were set *a priori* at *P*-value less than 0.05. Practical significance was gauged by evaluating standardized beta coefficients of each of the statistically significant predictors. Parameters for the two-step modeling processes were estimated using the maximum likelihood method. Metric scales were developed by fixing the first indicator of each latent variable to 1.0. Models were developed with Analysis of Moment Structure (AMOS) software version 18. Construct reliability and average variance extracted were calculated manually by computing formulas developed by Fornell and Larcker [30] with Microsoft Excel 2010. University Institutional Review Board (IRB) approval was sought and obtained prior to collecting participant data.

## 3. Results

### 3.1. Descriptive Statistics of Sample and Child Behaviors

Participants (*n* = 224) were predominantly Caucasian (72%), married (70%) and unemployed/homemakers (49%), with a mean age of 33.2 years (SD = 6.8). Data on two child behaviors were collected from the sample using 24 hour, proxy recall. The possible range for the child physical activity behavior construct was 0 to 500 minutes of physical activity in the previous 24 hours. The desired mean value was a minimum of 60 minutes. The observed range was 0 to 400 minutes with a mean of 101.77 minutes and a standard deviation of 80.52 minutes. The possible range for the child screen time behavior construct was 0 to 1,000 minutes of screen time in the previous 24 hours. The desired mean value was a maximum of 120 minutes. The observed range was 0 to 800 minutes with a mean of 104.65 minutes and a standard deviation of 98.61 minutes.

### 3.2. Model One: Physical Activity Behavior

**Figure 1:**
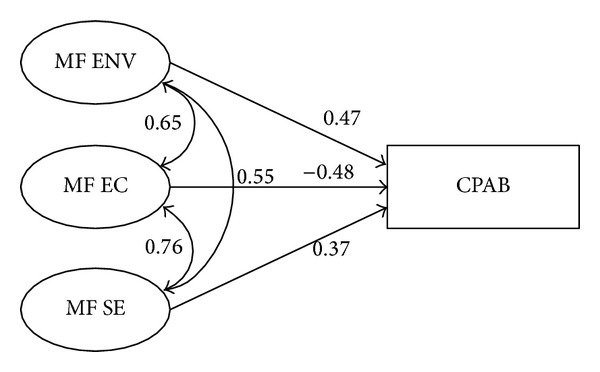
Maternal-facilitated child physical activity behavior structural model illustrating standardized regression weights for the sample of mothers (*n* = 206). Notes: CPAB is child physical activity behavior; MF ENV is maternal-facilitated environment; MF EC is maternal- facilitated emotional coping; MF SE is maternal-facilitated self-efficacy.

Mahalanobis *d*-squared values identified 18 impactful outliers; therefore, 206 samples were analyzed. Factor loadings, construct reliability values, and average variance extracted percentages satisfied the *a priori* thresholds. With the exception of one construct, child physical activity behavior (*r* = 0.67), all test-retest reliability coefficient values (*n* = 31) satisfied the 0.70 *a priori* reliability threshold. The final maternal-facilitated child physical activity behavior measurement model indicated reasonable fit to the data (*χ* ^2^ = 189.886, *df* = 92, *P* < 0.001; GFI = 0.893, AGFI = 0.842, NFI = 0.901, CFI = 0.946, and RMSEA = 0.072). Analysis of the structural model identified significant direct paths between maternal-facilitated environment (*P* < 0.001), emotional coping (*P* = 0.006), self-efficacy (*P* = 0.010), and child physical activity behavior. Specification indices indicated satisfactory fit for the final model (*χ* ^2^ = 51.268, *df* = 28, *P* = 0.005; GFI = 0.955, AGFI = 0.911, NFI = 0.940, CFI = 0.971, and RMSEA = 0.064). The model Chi-square test did not satisfy the *a priori* criteria of *P*-value greater than 0.05; however, the model Chi-square test is highly sensitive to sample sizes that exceed 200 observations [31]. Applying Kline's alternative to the model Chi-square test, model fit was satisfactory (51.268/28 = 1.831).  Table 1 summarizes parameter estimates and fit statistics of the structural model.  Figure 1 provides an illustration of the final structural model with standardized regression weights.

Collectively, the exogenous variables accounted for 22% of the variance in the child physical activity behavior construct. Emotional coping explained the greatest proportion of variance in the model (*β* = −0.479), followed by environment (*β* = 0.466) and self-efficacy (*β* = 0.372). Of the predictors, only emotional coping had a negative effect on physical activity behavior, which initially suggested that as emotional coping capacity increased physical activity quantity decreased. Further review of the construct's characteristics suggested that emotional coping acted as a suppressor variable within the model.

### 3.3. Suppression Variable Procedures

A series of four procedures were applied to determine if emotional coping was functioning as a suppressor variable [32, 33]. The first procedure involved examination of the correlation matrix to determine the relationship between the hypothesized suppressor, the other independent variables in the model, and the outcome variable. Suppressors are characterized by having a minimal direct relationship to the outcome variable, while being significantly correlated to other independent variables in the model [34]. The second procedure involved examining the impact of the hypothesized suppressor on the standardized beta weights of the other independent variables in the model. When included in a regression equation, suppressor variables increase the predictive validity of one or more other independent variables in the model. Concurrently, suppressors only control error variance for those independent variables whose regression weights increase due to its entry into the equation. The third procedure tested the possibility of multicollinearity. Detection of high multicollinearity within a regression model can be determined through examination of the correlational matrix and tolerance and variance inflation factor (VIF) values. Tolerance values less than 0.20 and VIF values greater than 5.0 are generally accepted cutoffs for indicating the presence of multicollinearity [33]. The final procedure included inspection of the suppressor's beta weight. Negative suppression occurs when the beta weight of the suppressor variable in the regression equation is the opposite sign from its correlation with the outcome variable.

Applying the first procedure verified that emotional coping had an insignificant, positive correlation with physical activity behavior (*r* = 0.088); yet, it had a statistically significant correlation with environment (*r* = 0.481) and self-efficacy (*r* = 0.601). The second procedure included examining the effect of the emotional coping variable upon removal from the structural model. Upon removing the hypothesized suppressor, results found that the *R* value of the physical activity behavior model decreased from 0.22 to 0.14, with the self-efficacy construct becoming statistically insignificant (*P* = 0.314) and the significance level of environment decreasing from a *P*-value less than 0.001 to *P*-value equal to 0.001. When the emotional coping variable was reinserted into the model, the total *R*-value once again increased to its pretest level of 0.22, the self-efficacy construct again became significant (*P* = 0.010), and the environment construct regained its former significance level (*P* < 0.001). Upon reintroduction of the suppressor, regression weights for self-efficacy increased from 0.091 to 0.372, while regression weights for environment increased from 0.318 to 0.466. These findings suggested that emotional coping primarily suppressed self-efficacy but also suppressed the environment construct. Next, multicollinearity was assessed. Of the significant variables in the final model, tolerance values were between 0.577 and 0.739 and VIF levels were between 1.354 and 1.732, indicating an absence of multicollinearity. Finally, the suppressor's beta weight within the regression equation was reviewed. Results found that, despite emotional coping's positive correlation with physical activity behavior, the construct displayed a negative beta weight within the regression equation, implying the status of a negative suppressor.

### 3.4. Model Two: Screen Time Behavior

**Figure 2:**
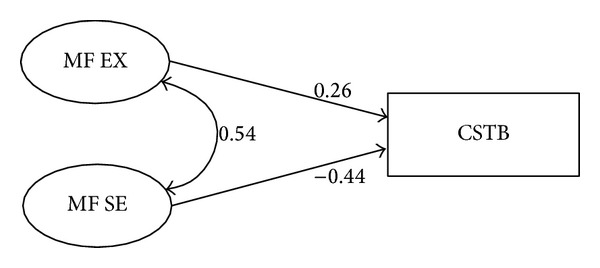
Maternal-facilitated child screen time behavior structural model illustrating standardized regression weights for the sample of mothers (*n* = 220). Notes: CSTB is child screen time behavior; MF EX is maternal-facilitated expectations; MF SE is maternal-facilitated self-efficacy.

Mahalanobis *d*-squared values identified four impactful outliers; therefore, 220 samples were analyzed. Examination of the initial measurement model found that the first environment indicator had a factor loading value of 0.24. As this fell below the 0.50 threshold, this variable was removed from the measurement model. The remaining factor loadings, construct reliability values, average variance extracted percentages, and test-retest reliability coefficient values satisfied the *a priori* thresholds. The final maternal-facilitated child screen time behavior measurement model indicated reasonable fit to the data (*χ* ^2^ = 254.11, *df* = 94, *P* < 0.001; GFI = 0.873, AGFI = 0.810, NFI = 0.904, CFI = 0.934, and RMSEA = 0.094). Analysis of the structural model identified significant direct paths between maternal-facilitated expectations (*P* = 0.002), self-efficacy (*P* < 0.001), and child screen time behavior. Specification indices indicated satisfactory fit for the final model (*χ* ^2^ = 20.216, *df* = 16, *P* = 0.211; GFI = 0.977, AGFI = 0.948, NFI = 0.980, CFI = 0.996, and RMSEA = 0.035).  Table 2 summarizes parameter estimates and fit statistics of the structural model.  Figure 2 provides an illustration of the final structural model with standardized regression weights.

Combined, emotional coping and environment accounted for 14% of the variance in the child screen time behavior construct. Inspection of the standardized beta weights of the significant variables found self-efficacy explained the greatest proportion of variance in the model (*β* = −0.479) followed by expectations (*β* = 0.466). The self-efficacy construct's negative beta weight suggested that as self-efficacy increased screen time behavior decreased. Among the predictors, only expectations had a positive effect on screen time behavior, which initially suggested that as expectations increased screen time quantity increased. Further review of the construct's characteristics suggested that expectations acted as a suppressor variable within the model.

The four procedures for suppressor variable detection were applied. Employing the first procedure verified that expectations had an insignificant, positive correlation with screen time behavior (*r* = 0.029); yet, it had a statistically significant correlation with self-efficacy (*r* = 0.491). The second procedure included examining the effect of the expectations variable upon removal from the structural model. Upon removing the hypothesized suppressor, results found that the *R*-value of the screen time behavior model decreased from 0.14 to 0.08, with the self-efficacy construct retaining its statistical significance in the model (*P* < 0.001). When the expectations variable was re-inserted into the model, the total *R*-value once again increased to its pretest level of 0.14. Upon reintroduction of the suppressor, standardized regression weights for self-efficacy increased from −0.279 to −0.432. Next, multicollinearity was assessed. Of the significant variables in the final model, tolerance values were between 0.599 and 0.690 and VIF levels were between 1.449 and 1.670, indicating an absence of multicollinearity. Finally, the suppressor's beta weight within the regression equation was reviewed. Results found that expectation's positive correlation with screen time behavior was retained within the regression equation, implying the status of a classical suppressor.

## 4. Discussion

Suppressor variables are variables that suppress or control for error variance in one or more independent variables in a model [34, 35]. Through this action, suppressor variables unleash the latent predictive power of one or more independent variables in the regression equation, thereby bolstering their beta weight(s) [32]. The term suppressor may initially seem obstructive to a model development; however, researchers have identified three primary advantages of retaining suppressor variables in multiple regressions: (1) increased accuracy of regression coefficients associated with independent variables, (2) improved predictive validity of a model, and (3) enhanced accuracy of a theory building [36].

Researchers have categorized three types of suppression effects in multiple correlations [36, 37]. Classical suppression occurs when the variable in question has a zero or minimal correlation with the dependent variable; yet, it significantly correlates with one or more independent variables in the model [38]. Negative suppression occurs when the beta weight of the suppressor variable in the regression equation is the opposite sign from its correlation with the outcome variable [39]. Reciprocal suppression occurs if both the suppressor and predictor are positively correlated with the outcome variable, yet negatively correlated with one another [40].

An important factor when interpreting suppressor variables is refrainment from the assumption that the negative beta weight of a suppressor implies a negative direct effect on the outcome variable; instead, the suppressor should be viewed as clearing out criterion-irrelevant variance in one or more other predictor variables [41]. To understand how suppressors function from an applied perspective, it is helpful to consider the case published by Horst [42], who was one of the first researchers to publish the suppressor concept. Horst described a study conducted during World War II that sought to predict pilot success in a training program. Measures of mechanical, numerical, spatial, and verbal ability were collected and correlated with training success. Results found that each of the variables, aside from verbal ability, was positively correlated with pilot success. Conversely, verbal ability had a near-zero correlation with pilot success yet was correlated with the other three predictors. Paradoxically, when verbal ability was entered into the regression equation the model's predictive capacity increased, despite verbal ability having insignificant correlation with pilot training. Horst's deduction was that verbal ability was required to read the directions and items included on the tests measuring the other three abilities. As such, verbal ability was acting as a suppressor by removing irrelevant variance from the other predictors, which assisted in clarifying their role in the model. As Horst [42] concluded, “To include the verbal score with a negative weight served to suppress or subtract irrelevant ability and to discount the scores of those who did well on the test simply because of their verbal ability rather than because of abilities required for success in pilot training (p. 355).”

In the context of the physical activity model, emotional coping was significantly correlated with self-efficacy and environment, which, in turn, were significantly related to physical activity behavior in children. In effect, emotional coping, despite having a minimal direct bivariate relationship with physical activity behavior in children, removed error variance from the self-efficacy and environment constructs. Thus, when included in the model, the emotional coping suppressor purified the role of self-efficacy and environment as predictors of child physical activity behavior. In practical terms, the suppression effect of the emotional coping construct suggests that emotional coping abilities are required to actualize self-efficacy and, to some extent, the home environment for increasing maternal-facilitated physical activity behavior in children. In terms of intervention design, emotional coping should be sufficiently developed in mothers prior to attempting to build self-efficacy or improve the home environment for increasing child physical activity. Results of the screen time model suggested that expectations for screen time behavior suppress criterion-irrelevant variance in the self-efficacy construct. The suppression effect of this variable implies that expectations for the reduction of screen time behavior in children are required to actualize self-efficacy for decreasing this sedentary behavior in children. From an intervention perspective, expectations should be sufficiently developed in mothers prior to attempting to build self-efficacy for decreasing child screen time behavior.

## 5. Conclusions

The purpose of this study was to model maternal-facilitated, SCT predictors of child physical activity and screen time. The intention of these modeling efforts was to provide design, implementation, and evaluation direction for primary prevention, family- and home-based, pediatric obesity interventions. Unexpectedly, results found that emotional coping and expectations acted as suppressor variables in predicting the maternal-facilitated physical activity and screen time behaviors of children. Other researchers have noted suppressor variables present in SCT models [43–45]; however, this study appears to be the first to identify suppressor variables in SCT models examining pediatric obesity within the context of the family and home environment. From an application orientation, suppressor variables can inform essential groundwork required to fully actualize predictors of a given behavior. From a programmatic perspective, behavioral prediction models are often limited in the fact that they can only provide a recommendation of what constructs to reify in an intervention. Suppressor effects can supplement intervention design recommendations by providing a suggested ordering to theoretical construct integration within an intervention.

Based on the models specified in this study, it is recommended to target the suppressor variables at the outset of intervention implementation. Specifically, to improve child physical activity behavior from a maternally-oriented, family and home-based perspective, the present research suggests, first, to develop emotional coping skills within mothers. Once sufficient levels of emotional coping skills are acquired, self-efficacy and environment for physical activity behavior should be developed. To reduce screen time within a maternally-oriented, family and home-based context, the current research suggests developing expectations for reduction of screen time in mothers prior to reifying self-efficacy for this behavior. Future research should test interventions for developing these constructs in mothers.

Although fit statistics suggested satisfactory reliability and validity of the structural equation models, the results of this research should be considered in light of several limitations. Data for this study were collected from a convenience sample of mothers with children between four and six years of age. As such, the results cannot be generalized to all mothers with children in this age range. The cross-sectional nature of the data restricts the ability to establish causal relationships between the variables. From a measurement perspective, the behavioral data were collected through 24 hour, maternal-proxy self-report. Consequently, the precision of the specified models was limited to the self-reporting accuracy and honesty of the participants. Measurement of the behaviors could be improved with accelerometers and screen time monitors with data collected in a longitudinal fashion. Finally, there are also limitations inherent to the design of this study. Primarily, the data only captured one demographic (mothers) and one environment (home of the child) hypothesized to contribute to the prevention of pediatric obesity. Pediatric obesity is a complex health issue that is impacted by multiple social and environmental variables. Therefore, the current models are inadequate to fully capture all antecedents of pediatric obesity, even if ideal measurement were granted. To the best of the author's knowledge, this is the first study to identify suppressor variables in SCT models examining pediatric obesity within the context of the family and home environment. Subsequently, the findings of this research could be strengthened by reproducing the study with new samples. In particular, it would be beneficial if the identified suppressor effects were confirmed or refuted through similar modeling endeavors.

## Figures and Tables

**Table 1:** Parameter estimates for the trimmed maternal-facilitated child physical activity behavior structural model for the sample of mothers (*n* = 206).

Variables	*B*	SE *B*	*β*	*P*
CPAB ← MF ENV	2.751	0.709	0.466	<0.001
CPAB ← MF SE	2.637	1.028	0.372	0.010
CPAB ← MF EC	−3.115	1.132	−0.479	0.006

Notes: CPAB: child physical activity behavior; MF ENV: maternal-facilitated environment; MF SE: maternal-facilitated self-efficacy; MF EC: maternal-facilitated emotional coping.

*χ* ^2^ = 51.268, *df* = 28, *P* = 0.005; GFI = 0.955, AGFI = 0.911, NFI = 0.940, CFI = 0.971, and RMSEA = 0.064.

*R* ^2^ value of CPAB for final specified model is 0.22.

**Table 2:** Parameter estimates for the trimmed maternal-facilitated child screen time behavior structural model for the sample of mothers (*n* = 220).

Variables	B	SE B	β	P
CSTB ← MF EX	0.216	0.069	0.262	0.002
CSTB ← MF SE	−2.481	0.510	−0.443	<0.001

Notes: CSTB: child screen time behavior; MF EX: maternal-facilitated expectations; MF SE: maternal-facilitated self-efficacy.

*χ* ^2^ = 20.216, *df* = 16, *P* = 0.211; GFI = 0.977, AGFI = 0.948, NFI = 0.980, CFI = 996, and RMSEA = 0.035.

*R* ^2^ value of PC STB for final specified model is 0.14.
